# Biohydrogenation of 22:6*n*-3 by *Butyrivibrio proteoclasticus* P18

**DOI:** 10.1186/s12866-016-0720-9

**Published:** 2016-06-10

**Authors:** Jeyamalar Jeyanathan, Marlene Escobar, Robert John Wallace, Veerle Fievez, Bruno Vlaeminck

**Affiliations:** Laboratory for Animal Nutrition and Animal Product Quality, Ghent University, Proefhoevestraat 10, 9090 Melle, Belgium; Rowett Institute of Nutrition and Health, University of Aberdeen, Bucksburn, Aberdeen, AB21 9SB UK

**Keywords:** 22:6*n*-3, Biohydrogenation, *Butyrivibrio*, Rumen fluid, *In vitro*, VFA

## Abstract

**Background:**

Rumen microbes metabolize 22:6*n*-3. However, pathways of 22:6*n*-3 biohydrogenation and ruminal microbes involved in this process are not known. In this study, we examine the ability of the well-known rumen biohydrogenating bacteria, *Butyrivibrio fibrisolvens* D1 and *Butyrivibrio proteoclasticus* P18, to hydrogenate 22:6*n*-3.

**Results:**

*Butyrivibrio fibrisolvens* D1 failed to hydrogenate 22:6*n*-3 (0.5 to 32 μg/mL) in growth medium containing autoclaved ruminal fluid that either had or had not been centrifuged. Growth of *B. fibrisolvens* was delayed at the higher 22:6*n*-3 concentrations; however, total volatile fatty acid production was not affected. *Butyrivibrio proteoclasticus* P18 hydrogenated 22:6*n*-3 in growth medium containing autoclaved ruminal fluid that either had or had not been centrifuged. Biohydrogenation only started when volatile fatty acid production or growth of *B. proteoclasticus* P18 had been initiated, which might suggest that growth or metabolic activity is a prerequisite for the metabolism of 22:6*n*-3. The amount of 22:6*n*-3 hydrogenated was quantitatively recovered in several intermediate products eluting on the gas chromatogram between 22:6*n*-3 and 22:0. Formation of neither 22:0 nor 22:6 conjugated fatty acids was observed during 22:6*n*-3 metabolism. Extensive metabolism was observed at lower initial concentrations of 22:6*n*-3 (5, 10 and 20 μg/mL) whereas increasing concentrations of 22:6*n*-3 (40 and 80 μg/mL) inhibited its metabolism. Stearic acid formation (18:0) from 18:2*n*-6 by *B. proteoclasticus* P18 was retarded, but not completely inhibited, in the presence of 22:6*n*-3 and this effect was dependent on 22:6*n*-3 concentration.

**Conclusions:**

For the first time, our study identified ruminal bacteria with the ability to hydrogenate 22:6*n*-3. The gradual appearance of intermediates indicates that biohydrogenation of 22:6*n*-3 by *B. proteoclasticus* P18 occurs by pathways of isomerization and hydrogenation resulting in a variety of unsaturated 22 carbon fatty acids. During the simultaneous presence of 18:2*n*-6 and 22:6*n*-3, *B. proteoclasticus* P18 initiated 22:6*n*-3 metabolism before converting 18:1 isomers into 18:0.

## Background

Docosahexaenoic acid (22:6*n*-3) is a poly-unsaturated fatty acid (PUFA) that has been associated with physiological benefits in many species, including human and dairy cows. The amount of 22:6*n*-3 available for absorption in the small intestine can be increased by intake of marine products (e.g. fish oil, marine algae). However, this is not straightforward in ruminants as extensive biohydrogenation in the rumen leaves little 22:6*n*-3 for absorption.

Biohydrogenation of PUFA is one of the important microbial processes in the rumen that has a major influence on FA composition of meat and milk. It is well documented that ruminal bacteria are responsible for most of the biohydrogenation process in the rumen [1], and it is thought to be a detoxification mechanism as PUFA are more toxic than saturated FA [2]. Metabolism of linoleic (18:2*n*-6) and linolenic (18:3*n*-3) acids is well studied *in vivo* and *in vitro* and involves an initial isomerization step to yield a FA with a conjugated double bond (a pair of double bonds separated by one single bond). *In vitro* studies with mixed and pure rumen bacteria helped to construct the detailed biohydrogenation pathways of 18:2*n*-6 and 18:3*n*-3, and the identification of bacterial species involved in this process [3–5]. However, information on the biohydrogenation of 22:6*n*-3 is still lacking. Several reports show extensive metabolism of 22:6*n*-3 *in vivo* [6, 7] and *in vitro* with mixed rumen cultures [8, 9]. However, none of the studies identified bacterial species responsible for its metabolism. Maia et al. [10] investigated PUFA metabolism of 26 predominant rumen bacterial species and found none of them able to metabolize 22:6*n*-3. Sakurama et al. [11] screened about 100 strains of anaerobic bacteria including some ruminal bacteria and found none of them metabolized 22:6*n*-3. Pure culture studies focusing on the main rumen hydrogenating bacteria, *Butyrivibrio fibrisolvens* and *Butyrivibrio proteoclasticus* also failed to successfully induce 22:6*n*-3 hydrogenation [10].

The aim of this study was to examine the metabolism of 22:6*n*-3 by the known biohydrogenating ruminal bacteria, *B. fibrisolvens* and *B. proteoclasticus*. To our knowledge, in previous experiments these bacterial species have been exposed to a single concentration of 22:6*n*-3 (50 μg/mL) which may be toxic for them [10]. We used lower concentrations of 22:6*n*-3 in our experiments. Additionally, we modified the standard *Butyrivibrio* growth medium in an attempt to promote biohydrogenation. Experiments 1–5 were conducted using the growth medium containing autoclaved-uncentrifuged rumen fluid and experiments 6−8 were conducted using the growth medium containing autoclaved-centrifuged rumen fluid (Table 1).

**Table 1:** Overview of the *in vitro* experiments conducted in this study

Exp.	Autoclaved-rumen fluid/tube (%)	Bacteria^a^	22:6*n*-3 (μg/mL)	Gas phase^b^	Incubation period (h)
	Uncentrifuged				
1	20 and 50	B. fibri	20	CO_2_	0 and 48
2	50	B. proteo	20	CO_2_, H_2_ and N_2_	0 and 48
3	50	B. proteo	20	H_2_	0, 2, 4, 8, 12, 24 and 48
4	50	B. proteo	5, 10, 40 and 80	H_2_	0 and 48
5^c^	50	B. proteo	10 and 40	H_2_	0, 2, 4, 8, 12, 24 and 48
	Centrifuged				
6	20 and 50	B. fibri	0.5, 1, 2, 4, 8, 16 and 32	CO_2_	0 and 48
7	20	B. proteo	20	H_2_	0 and 24
8	20	B. proteo	20	H_2_	0, 2, 4, 8, 12 and 24

^a^Bacteria used in the experiment -B. fibri*: Butyrivibrio fibrisolvens* D1; B. proteo: *Butyrivibrio proteoclasticus* P18

^b^headspace gas phase

^c^The growth medium used in experiment 5 contained 40 μg/mL of 18:2*n*-6

## Results

### 22:6*n*-3 metabolism by *B. fibrisolvens* D1

Both autoclaved-centrifuged and -uncentrifuged rumen fluid (Exp. 1 and 6), did not result in 22:6*n*-3 metabolism at any concentration. The effect of various concentrations of 22:6*n*-3 on growth, fermentation and biohydrogenation ability of *B. fibrisolvens* D1 (Exp. 6) in media containing 20 % (v/v) autoclaved-centrifuged rumen fluid are summarized in Table 2. No growth was observed till 48 h with the highest concentration of 22:6*n*-3 (32 μg/mL), whereas a considerably shorter lag phase (4 h) was observed with the same concentration of 18:2*n*-6 in the same medium. Optical density (OD_600_) measured at stationary phase was lower (*P* < 0.05) at the concentration of 16 μg/mL compared to the lower concentrations (0 and 8 μg/mL). Total volatile fatty acid (VFA) production and VFA profile were not affected (*P* > 0.05) by the 22:6*n*-3 concentrations. The main fermentation products included butyrate and small amounts of acetate (about 96 % and 4 % of molar concentration of VFA respectively).

**Table 2:** Effects of different concentrations of 22:6*n*-3 on growth, VFA production and biohydrogenation by *Butyrivibrio fibrisolvens* D1

	Concentration of 22:6*n*-3 (μg/mL)			
	0	8	16	32
Lag phase (h)	< 3	< 3	4-5	> 48
OD_600_^c^	1.13 ± 0.07^a^	1.22 ± 0.10^a^	1.04 ± 0.07^b^	ND^d^
Total VFA (μmol/tube)	93.0 ± 3.3	94.0 ± 6.2	94.2 ± 4.9	ND
Biohydrogenation	No	No	No	No

^c^Increase in OD_600_ after 48 h of incubation compared to initial OD_600_ at 0 h

^d^ND- Not determined as growth was not started till 48 h

Means with different superscripts in OD_600_ (a and b) are significantly different (*P* < 0.05)

### 22:6*n*-3 metabolism by *B. proteoclasticus* P18

In the growth medium containing 50 % (v/v) autoclaved-uncentrifuged rumen fluid, *B. proteoclasticus* P18 hydrogenated 22:6*n*-3 (Exp. 2). At the concentrations of 20 μg/mL (0.2 mg/tube), most of the 22:6*n*-3 was metabolized, leaving little residual 22:6*n*-3 (0.005 ± 0.003 mg/tube) after 48 h of incubation. The type of headspace gas had no influence on the residual amount of 22:6*n*-3. Total VFA production and VFA profile were not affected (*P* > 0.05) by different gas phases (data not shown). The main fermentation products included butyrate and acetate (about 70 % and 30 % of molar concentration of VFA respectively).

### Effect of length of incubation period on 22:6*n*-3 metabolism by *B. proteoclasticus* P18

**Fig. 1:**
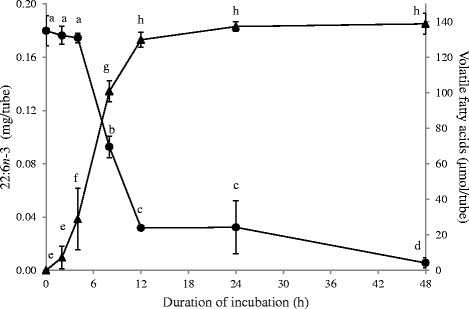
Metabolism of 22:6*n*-3 and VFA accumulation at different time points by *Butyrivibrio proteoclasticus* P18. Growth medium included 50 % (v/v) of autoclaved-uncentrifuged rumen fluid. Hydrogen was used as the headspace gas. ● Residual 22:6*n*-3 in the tube and ▲ VFA accumulated. Results are means and SD from 2 biological replicates, each of which had 2 analytical replicates. For residual 22:6*n*-3 and accumulated VFA, data points with different letters are significantly different (*P* < 0.05)

The extent of 22:6*n*-3 metabolism in media containing 50 % (v/v) autoclaved-uncentrifuged rumen fluid at different time points (Exp. 3) is shown in Fig. 1 along with the VFA production at the respective time points. Metabolism of 22:6*n*-3 (20 μg/mL) initiated after 4 h and 80 % of 22:6*n*-3 was hydrogenated between 4 h and 12 h of incubation. Accumulation of VFA initiated prior to the start of 22:6*n*-3 metabolism and 93 % of VFA were produced within the first 12 h of the incubation. Both the rate of 22:6*n*-3 disappearance and VFA production slowed down after 12 h of incubation. Significant VFA production was not observed after 12 h, whereas hydrogenation of 22:6*n*-3 slowly continued. As a result, the amount of 22:6*n*-3 recovered after 48 h was lower (*P* < 0.05) than the amount recovered after 12 and 24 h of incubation.

**Fig. 2:**
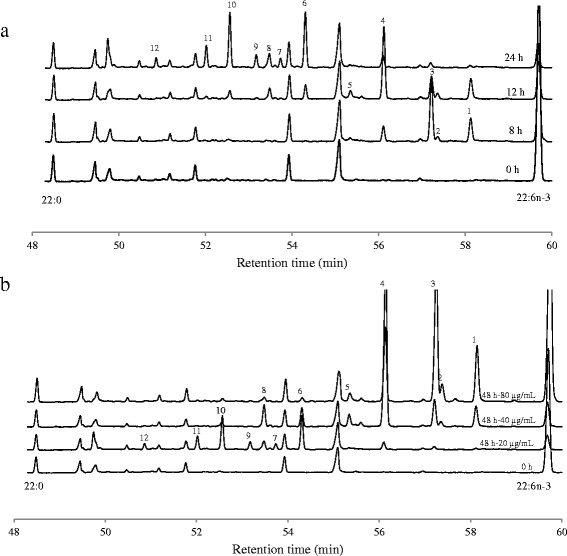
Partial chromatogram with peaks representing fatty acids from 22:0 to 22:6*n*-3. These chromatograms obtained from (**a**) 0, 8, 12 and 24 h incubations with 22:6*n*-3 (20 μg/mL) and (**b**) 48 h incubations with 22:6n-3 (20, 40 and 80 μg/mL). In both cases *Butyrivibrio proteoclasticus* P18 had grown in the media containing 50 % (v/v) autoclaved- uncentrifuged rumen fluid. Peaks visible at 0 h represent the fatty acids present in the rumen fluid and peaks numbers (1–12) represent new peaks formed during the 22:6*n*-3 metabolism

Detailed analysis of chromatograms did not provide evidence of 22:0 formations during metabolism of 22:6*n*-3. Peaks appearance at different time points did not show accumulation of conjugated 22:6 products. The intermediates produced during the 48 h incubation comprised about 12 different FA (Table 3) and characteristic ion fragments are presented in Table 4. During the early hours of incubation (<8 h), 4 new peaks (i.e. peaks which were not present in the 0 h incubation) appeared (Fig. 2a: peaks 1-4). Of these, peak 3 was the most abundant and the molecular ion at m/z = 383 and the loss of the terminal methyl group (m/z = 368) confirmed the docosapentaenoic acid structure. The odd numbered ion fragment at m/z = 153 is an indication for the location of a Δ5 double bond [12]. Gaps of 12 amu between m/z = 208 and 220; 248 and 260; 288 and 300 and between 328 and 340 confirmed the location of ethylenic double bonds in position Δ10, Δ13, Δ16 and Δ19 (Table 4). Peak 1 showed the same retention time as 22:5*n*-3 present in the FAME standard and ion fragments at 168 and 180, 208 and 220, 248 and 260, 288 and 300, and 328 and 340 allowed us to identify this peak as 22:5*n*-3 [13]. A final docosapentaenoic acid (peak 2) was identified as Δ4, Δ10, Δ13, Δ16, Δ19-22:5 based on the molecular ion at m/z = 383, gaps of 12 amu between m/z = 208 and 220; 248 and 260; 288 and 300 and between 328 and 340 and the double bond in position 4 is defined by the fingerprint ion at m/z = 139 and 152 [14]. One docosatetraenoic acid was identified as Δ10, Δ13, Δ16, Δ19-22:4 (peak 4) based on the molecular ion at m/z = 385 and gaps of 12 amu between m/z = 210 and 222; 250 and 262; 290 and 302 and between 330 and 342. During the further incubation, the abundance of these peaks gradually decreased and were accompanied with the appearance of four 22:3 FA (peaks 5-8: Fig. 2a) and four 22:2 FA (peaks 9-12: Fig. 2a). Peaks 5 and 7 showed a similar mass spectra and were identified as Δ13, Δ16, Δ20-22:3. Ion fragments separated by 12 amu gaps located the double bonds at Δ13, Δ16 and Δ20 with a prominent ion at m/z 332 confirming the presence of allylic bonds at Δ16 and Δ20. Peak 6 showed the same retention time as 22:3n-3 present in the FAME standard and double bond positions were confirmed by ion fragments separated by 12 amu gaps (Table 4). Peak 8 was identified as Δ11, Δ16, Δ19-22:3 based on ion fragments at 224 and 236, 292 and 304, and 332 and 344. Peaks 9 and 11 showed a similar mass spectra and were identified as Δ16, Δ20-22:2. Ion fragments separated by 12 amu gaps located the double bonds at Δ16 and Δ20 (Table 4) with a prominent ion at m/z 334 confirming the presence of allylic bonds at Δ16 and Δ20. Peak 10 was Δ16, Δ19-22:2 (ion fragments separated by 12 amu gaps at 294 and 306, and 334 and 346) and the prominent ion at m/z 306 combined ion fragments separated by 12 amu gaps at 266 and 278, and 320 and 332 allowed us to identify peak 12 as Δ14, Δ18-22:2.

**Table 3:** Amount of metabolized 22:6*n*-3 (μg/tube) recovered in FAME peaks. Incubation was performed in growth medium containing autoclaved-uncentrifuged rumen fluid (50 % v/v) in the presence of 22:6*n*-3 (20 μg/mL) with *Butyrivibrio proteoclasticus* P18. Tubes were withdrawn at 0, 2, 4, 8, 12, 24 and 48 h incubation to study the extent of 22:6*n*-3 biohydrogenation. Peak numbers correspond to peaks in Fig. 2

Peaks		Incubation duration (h)				
		4	8	12	24	48
1	Δ7, Δ10, Δ13, Δ16, Δ19-22:5	2.18 ± 0.66	16.88 ± 0.94	16.38 ± 2.09	3.39 ± 1.71	2.93 ± 1.04
2	Δ4, Δ10, Δ13, Δ16, Δ19-22:5	-	3.36 ± 0.38	4.19 ± 1.04	1.07 ± 0.34	0.43 ± 0.58
3	Δ5, Δ10, Δ13, Δ16, Δ19-22:5	3.92 ± 1.29	44.38 ± 1.59	16.60 ± 5.80	7.40 ± 3.51	3.68 ± 2.42
4	Δ10, Δ13, Δ16, Δ19-22:4		9.42 ± 2.55	55.40 ± 3.00	11.27 ± 2.34	19.69 ± 2.41
5	Δ13, Δ16, Δ20-22:3		3.12 ± 0.70	8.17 ± 1.99	1.73 ± 0.11	3.63 ± 1.62
6	Δ13, Δ16, Δ19-22:3		-	9.91 ± 1.90	33.05 ± 4.66	36.02 ± 5.88
7	Δ13, Δ16, Δ20-22:3		-	0.69 ± 0.86	4.91 ± 0.38	7.24 ± 0.49
8	Δ11, Δ16, Δ19-22:3		-	9.10 ± 0.99	10.69 ± 1.66	8.77 ± 1.22
9	Δ16, Δ20-22:2		-	0.72 ± 0.90	7.45 ± 0.49	10.75 ± 0.76
10	Δ16, Δ19-22:2		-	6.31 ± 1.67	31.24 ± 2.96	37.31 ± 4.61
11	Δ16, Δ20-22:2		-	1.34 ± 0.93	11.75 ± 1.25	16.69 ± 1.46
12	Δ14, Δ18-22:2		-	-	5.03 ± 0.48	7.49 ± 0.56

**Table 4:** Characteristic ion fragments recorded during gas-chromatography mass-spectrometry analysis of 4,4-dimethyloxazoline derivatives of newly formed fatty acids during biohydrogenation of 22:6*n*-3 by *Butyrivirbio proteoclasticus*. Peak numbers correspond to peaks in Fig. 2

Peak	Fatty acid	Characteristic ion fragments (m/z, relative intensity)
1	Δ7, Δ10, Δ13, Δ16, Δ19-22:5	113 (64), 126 (100), 168 (9), 180 (24), 194 (21), 208 (11), 220 (6), 234 (12), 248 (16), 260 (6), 274 (12), 288 (6), 300 (5), 314 (10), 328 (3), 340 (2), 354 (2), 368 (4), 383 (9)
2	Δ4, Δ10, Δ13, Δ16, Δ19-22:5	113 (100), 126 (17), 139 (15), 152 (49), 166 (52), 180 (15), 194 (25), 208 (4), 220 (5), 234 (14), 248 (6), 260 (7), 274 (1), 288 (8), 300 (4), 314 (12), 328 (3), 340 (2), 354 (4), 368 (4), 383 (11)
3	Δ5, Δ10, Δ13, Δ16, Δ19-22:5	113 (100), 126 (21), 153 (18), 166 (4), 180 (14), 194 (9), 208 (1), 220 (3), 234 (8), 248 (2), 260 (1), 274 (3), 288 (2), 300 (1), 314 (4), 328 (1), 340 (1), 354 (1), 368 (2), 383 (3)
4	Δ10, Δ13, Δ16, Δ19-22:4	113 (88), 126 (100), 168 (11), 182 (10), 196 (13), 210 (5), 222 (4), 236 (6), 250 (13), 262 (4), 276 (12), 290 (24), 302 (8), 316 (25), 330 (8), 342 (5), 356 (6), 370 (8), 385 (28)
5/7	Δ13, Δ16, Δ20-22:3	113 (64), 126 (57), 168 (9), 182 (6), 196 (4), 210 (4), 224 (4), 238 (7), 252 (3), 264 (1), 278 (8), 292 (7), 304 (3), 318 (3), 332 (100), 346 (2), 358 (3), 372 (8), 387 (17)
6	Δ13, Δ16, Δ19-22:3	113 (100), 126 (100), 168 (14), 182 (11), 196 (8), 210 (7), 224 (7), 238 (10), 252 (4), 264 (2), 278 (7), 292 (12), 304 (5), 318 (22), 332 (16), 344 (8), 358 (13), 372 (15), 387 (42)
8	Δ11, Δ16, Δ19-22:3	113 (80), 126 (100), 168 (18), 182 (11), 196 (9), 210 (11), 224 (7), 236 (4), 250 (3), 264 (9), 278 (45), 292 (2), 304 (5), 318 (14), 332 (6), 344 (5), 358 (3), 372 (15), 387 (21)
9/11	Δ16, Δ20-22:2	113 (100), 126 (56), 168 (11), 182 (7), 196 (4), 210 (3), 224 (3), 238 (6), 252 (3), 266 (4), 280 (10), 294 (8), 306 (1), 320 (2), 334 (75), 348 (2), 360 (2), 374 (6), 389 (11)
10	Δ16, Δ19-22:2	113 (100), 126 (69), 168 (12), 182 (8), 196 (5), 210 (4), 224 (6), 238 (8), 252 (5), 266 (6), 280 (10), 294 (3), 306 (2), 320 (13), 334 (11), 346 (9), 360 (10), 374 (19), 389 (34)
12	Δ14, Δ18-22:2	113 (100), 126 (83), 168 (14), 182 (15), 196 (9), 210 (6), 224 (7), 238 (13), 252 (7), 266 (7), 278 (3), 292 (14), 306 1(80), 320 (<1), 332 (5), 346 (12), 360 (25), 374 (14), 389 (26)

**Fig. 3:**
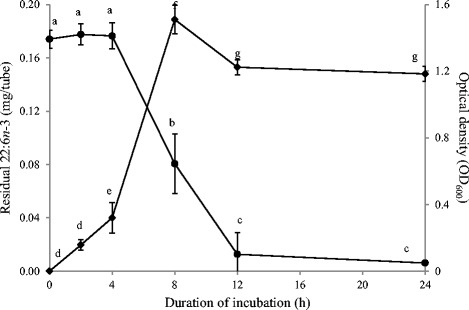
Metabolism of 22:6*n*-3 and optical density measurements at different time points by *Butyrivibrio proteoclasticus* P18. Growth medium included 20 % (v/v) of autoclaved-centrifuged rumen fluid. Hydrogen was used as the headspace gas. ● Residual 22:6n-3 in the tube ♦ Optical density (OD_600_) measured at respective time points. Results are means and SD from 3 replicates. For residual 22:6*n*-3 and OD_600_ measured, data points with different letters are significantly different (*P* < 0.05)

In experiments conducted in media containing autoclaved-centrifuged rumen fluid (20 % v/v), *B. proteoclasticus* hydrogenated 22:6*n*-3 at the concentration of 20 μg/mL (Exp. 7 and 8). After 24 h of incubation, little residual 22:6*n*-3 was left in the tube (0.006 ± 0.0003 mg/tube). Figure 3 shows the kinetics of 22:6*n*-3 disappearance along with OD_600_ measured at the respective time points (Exp. 8). Metabolism of 22:6*n*-3 initiated after 4 h and 93 % of 22:6*n*-3 was hydrogenated between 4 h and 12 h of incubation. Growth of *B. proteoclasticus* (measured as the increase in the OD_600_) initiated prior to the start of 22:6*n*-3 metabolism and exponentially increased between 4 h and 12 h. The products formed during the biohydrogenation of 22:6*n*-3 (Table 5) were identical as those described before (Exp. 3).

**Table 5:** Amount of metabolized 22:6*n*-3 (μg/tube) recovered in FAME peaks. Incubation was performed in growth medium containing autoclaved-centrifuged rumen fluid (20 % v/v) in the presence of 22:6*n*-3 (20 μg/mL) with *Butyrivibrio proteoclasticus* P18. Tubes were withdrawn at 0, 2, 4, 8, 12 and 24 h incubation to study the extent of 22:6*n*-3 biohydrogenation. Peak numbers correspond to peaks in Fig. 2

Peaks		Incubation duration (h)			
		4	8	12	24
1	Δ7, Δ10, Δ13, Δ16, Δ19-22:5	5.51 ± 0.59	19.55 ± 2.82	13.72 ± 3.93	-
2	Δ4, Δ10, Δ13, Δ16, Δ19-22:5	-	3.68 ± 1.22	5.21 ± 3.88	-
3	Δ5, Δ10, Δ13, Δ16, Δ19-22:5	4.41 ± 1.10	53.32 ± 8.58	28.37 ± 39.9	-
4	Δ10, Δ13, Δ16, Δ19-22:4		11.34 ± 3.05	91.90 ± 40.6	26.41 ± 36.1
5	Δ13, Δ16, Δ20-22:3		6.89 ± 1.25	11.86 ± 3.27	9.34 ± 5.26
6	Δ13, Δ16, Δ19-22:3		-	10.26 ± 5.80	38.69 ± 3.97
7	Δ13, Δ16, Δ20-22:3		-	0.36 ± 0.32	6.75 ± 5.59
8	Δ11, Δ16, Δ19-22:3		-	10.10 ± 6.52	15.73 ± 16.6
9	Δ16, Δ20-22:2		-	0.34 ± 0.30	7.36 ± 7.36
10	Δ16, Δ19-22:2		-	1.38 ± 1.21	26.68 ± 11.4
11	Δ16, Δ20-22:2		-	-	10.09 ± 10.8
12	Δ14, Δ18-22:2		-	-	6.69 ± 5.96

### Effect of initial concentration of 22:6*n*-3 on 22:6*n*-3 metabolism by *B. proteoclasticus* P18

**Fig. 4:**
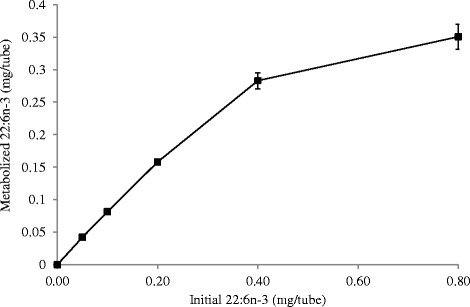
Effect of initial concentration of 22:6*n*-3 (5 - 80 μg/mL) on its metabolism by *Butyrivibrio proteoclasticus* P18. Incubations were performed in growth medium containing 50 % (v/v) of autoclaved-uncentrifuged rumen fluid for 48 h

The initial concentration of 22:6*n*-3 influenced the extent of its metabolism. Extensive metabolism of 22:6*n*-3 was observed at lower concentrations (5, 10 and 20 μg/mL). Addition of 5, 10 and 20 μg/ml 22:6*n*-3 resulted in the accumulation of 22:2 and 22:3 FA and represented > 80 % of 22:6*n*-3 that was metabolized during the 48 h incubation (Table 6). This implies that the initial 22:5 and 22:4 FA were further hydrogenated. An increase in the initial concentration of 22:6*n*-3 increased the amount of 22:6*n*-3 metabolized during 48 h incubation period, but only up to a concentration of about 40 μg/mL (Fig. 4). Although in absolute amounts, more 22:6*n*-3 disappeared at higher 22:6*n*-3 concentrations, analysis of the chromatograms showed that extensive biohydrogenation of intermediate products was inhibited (Table 6 and Fig. 2b). At the initial concentration of 40 μg/mL, the residual 22:6*n*-3 retrieved from the tubes after 48 h incubation was 9 % of the initial amount. Under this condition, 65 % of the metabolized 22:6*n*-3 accumulated as a single compound identified as Δ10, Δ13, Δ16, Δ19-22:4. Further conversion of this product seemed to be inhibited. When the initial 22:6*n*-3 concentration was increased to 80 μg/mL, the residual 22:6*n*-3 retrieved from the tubes after 48 h of incubation was 45 % of the initial amount and > 90 % of the metabolized 22:6*n*-3 accumulated as 22:4 and 22:5 FA. Subsequent biohydrogenation of these products seemed completely inhibited at this concentration.

**Table 6:** Amount of metabolized 22:6*n*-3 (μg/tube) recovered in FAME peaks. Incubation was performed in growth medium containing autoclaved-uncentrifuged rumen fluid (50 % v/v) in the presence of 22:6*n*-3 (5, 10, 20, 40 and 80 μg/mL) with *Butyrivibrio proteoclasticus* P18. Tubes were withdrawn at 48 h of incubation to study the extent of 22:6n-3 biohydrogenation. Peak numbers correspond to peaks in Fig. 2

		Initial concentration of 22:6*n*-3 (μg/tube)				
Peaks		50	100	200	400	800
1	Δ7, Δ10, Δ13, Δ16, Δ19-22:5	0.63 ± 0.75	1.15 ± 0.28	2.93 ± 1.04	25.14 ± 3.03	77.118 ± 1.30
2	Δ4, Δ10, Δ13, Δ16, Δ19-22:5	-	-	0.43 ± 0.58	5.08 ± 1.05	21.42 ± 0.69
3	Δ5, Δ10, Δ13, Δ16, Δ19-22:5	0.87 ± 1.13	1.11 ± 0.96	3.68 ± 2.42	29.16 ± 3.92	177.29 ± 5.04
4	Δ10, Δ13, Δ16, Δ19-22:4	5.26 ± 1.98	8.14 ± 2.11	19.69 ± 2.41	208.41 ± 14.7	78.81 ± 7.61
5	Δ13, Δ16, Δ20-22:3	0.41 ± 0.82	1.17 ± 0.82	3.63 ± 1.62	15.80 ± 2.34	12.05 ± 1.17
6	Δ13, Δ16, Δ19-22:3	5.82 ± 0.77	14.26 ± 0.62	36.02 ± 5.88	22.22 ± 3.63	3.48 ± 1.19
7	Δ13, Δ16, Δ20-22:3	2.84 ± 0.46	5.02 ± 0.20	7.24 ± 0.49	-	-
8	Δ11, Δ16, Δ19-22:3	2.33 ± 0.86	4.26 ± 0.66	8.77 ± 1.22	26.98 ± 3.47	5.61 ± 0.81
9	Δ16, Δ20-22:2	4.14 ± 0.53	7.14 ± 0.39	10.75 ± 0.76	-	-
10	Δ16, Δ19-22:2	7.81 ± 0.81	15.92 ± 0.87	37.31 ± 4.61	5.53 ± 1.24	3.09 ± 1.05
11	Δ16, Δ20-22:2	8.83 ± 0.63	12.94 ± 0.59	16.69 ± 1.46	-	-
12	Δ14, Δ18-22:2	3.34 ± 0.65	5.09 ± 0.35	7.49 ± 0.56	-	-

Total VFA production was not affected by the initial concentration of 22:6*n*-3 except for the highest concentration (80 μg/mL). At the highest initial concentration (80 μg/mL) VFA production after 48 h was higher (*P* < 0.05*)* compared to the lower concentrations (16.4 ± 0.5 and 15.1 ± 0.5 μmol/mL respectively), but the VFA profile was not affected (*P* > 0.05).

### Effect of presence of 22:6*n*-3 on 18:2*n*-6 metabolism by *B. proteoclasticus* P18

Table 7 shows the conjugated linoleic acids (CLA), vaccenic acid (VA; trans-11-18:1) and stearic acid (SA;18:0) formation from 40 μg/mL 18:2*n*-6 in presence of 0 (control), low (10 μg/mL) and high (40 μg/mL) 22:6*n*-3, along with VFA formation and residual 22:6*n*-3. The formation of CLA was observed as soon as inoculation was made. Although formation of CLA and VA were not affected (*P* > 0.05), conversion of VA into SA (18:0) was retarded (*P* > 0.05) in the presence of 22:6*n*-3 and this effect was dependent on 22:6*n*-3 concentration. Volatile fatty acids production was not initiated until all the 18:2*n*-6 had been metabolized and converted into VA regardless of presence of high or low 22:6*n*-3. Both 18:0 formation and 22:6*n*-3 metabolism began after the initiation of VFA production. In the control tubes, 18:0 formation started at about 4 h as soon as VFA production began and this conversion was rapid thereafter (Table 7). In the presence of low 22:6*n*-3 (10 μg/mL), *B. proteoclasticus* P18 initiated 22:6*n*-3 metabolism around 4 h, ahead of 18:0 formation (around 8 h of incubation). As a result, a small lag phase was observed in 18:0 formation after the initiation of VFA production. In presence of high 22:6*n*-3 (40 μg/mL), 18:0 formation was delayed to 12 h and this conversion was slower thereafter.

**Table 7:** Linoleic acid metabolism (mg/tube) in presence of 22:6*n*-3 by *B. proteoclasticus* P18. Incubation was performed in growth medium containing 40 μg/mL (0.4 mg/tube) of 18:2*n*-6 in presence of 0, 10 μg/mL (Low: 0.1 mg/tube) and 40 μg/mL (High: 0.4 mg/tube) 22:6*n*-3. Growth medium included 50 % (v/v) of autoclaved-uncentrifuged rumen fluid and H_2_ gas was the headspace gas. Value-presented in this table included the fatty acids and VFA of autoclaved-uncentrifuged rumen fluid used to prepare the growth medium

	Incubation duration (h)							
	22:6n-3	0	2	4	8	12	24	48
18:2*n*-6	0	0.27 ± 0.006^a^	0.09 ± 0.014^b^	0.08 ± 0.009^bc^	0.08 ± 0.002^bc^	0.09 ± 0.012^b^	0.06 ± 0.001^c^	0.05 ± 0.002^c^
(mg/tube)	Low	0.25 ± 0.007^a^	0.09 ± 0.001^bc^	0.09 ± 0.002^bc^	0.09 ± 0.001^bc^	0.10 ± 0.003^b^	0.07 ± 0.006^c^	0.06 ± 0.002^c^
	High	0.28 ± 0.099^a^	0.11 ± 0.002^b^	0.10 ± 0.011^bc^	0.10 ± 0.004^b^	0.10 ± 0.006^b^	0.07 ± 0.006^c^	0.06 ± 0.005^c^
CLA	0	0.07 ± 0.005^a^	0.02 ± 0.007^b^	0.00^b^	0.00^b^	0.00^b^	0.00^b^	0.00^b^
(mg/tube)	Low	0.10 ± 0.007^a*^	0.02 ± 0.004^b^	0.00^b^	0.00^b^	0.00^b^	0.00^b^	0.00^b^
	High	0.11 ± 0.073^a^	0.02 ± 0.003^b^	0.00^b^	0.00^b^	0.00^b^	0.00^b^	0.00^b^
VA	0	0.05 ± 0.005^a^	0.27 ± 0.026^b^	0.21 ± 0.011^c^	0.07 ± 0.003^d^	0.06 ± 0.005^d^	0.06 ± 0.003^d^	0.04 ± 0.008^a^
(mg/tube)	Low	0.05 ± 0.005^a^	0.30 ± 0.010^b*^	0.29 ± 0.015^c*^	0.10 ± 0.007^d*^	0.07 ± 0.003^e^	0.07 ± 0.004^e^	0.07 ± 0.004^e*^
	High	0.05 ± 0.026^a^	0.29 ± 0.005^b*^	0.28 ± 0.005^b*^	0.19 ± 0.005^c**^	0.11 ± 0.008^d*^	0.13 ± 0.010^d*^	0.10 ± 0.002^d**^
18:0	0	0.50 ± 0.011^a^	0.51 ± 0.041^a^	0.58 ± 0.014^b^	0.74 ± 0.056^c^	0.73 ± 0.020^c^	0.71 ± 0.016^c^	0.73 ± 0.019^c^
(mg/tube)	Low	0.53 ± 0.019^a^	0.53 ± 0.009^a^	0.53 ± 0.022^a*^	0.67 ± 0.019^b*^	0.69 ± 0.018^bc*^	0.72 ± 0.010^c^	0.73 ± 0.010^c^
	High	0.53 ± 0.006^a^	0.53 ± 0.003^a^	0.52 ± 0.017^a*^	0.54 ± 0.015^a**^	0.58 ± 0.015^b**^	0.65 ± 0.017^c*^	0.68 ± 0.007^c*^
VFA	0	434.1 ± 4.79^a^	436.4 ± 7.77^a^	469.6 ± 16.80^b^	541.7 ± 12.61^c^	545.7 ± 8.57^c^	585.2 ± 30.61^d^	567.6 ± 15.93^d^
(μmol/tube)	Low	434.0 ± 9.61^a^	437.7 ± 6.01^a^	460.6 ± 3.09^b^	527.9 ± 6.54^cd^	542.9 ± 4.34^de^	557.0 ± 7.23^e*^	551.4 ± 5.35^e^
	High	449.1 ± 16.6^a^	433.0 ± 8.91^a^	468.5 ± 21.71^b^	531.8 ± 8.25^c^	549.7 ± 6.14^cd^	562.1 ± 17.97^d*^	559.4 ± 2.82^d^
22:6*n*-3	Low	0.07 ± 0.005^a^	0.07 ± 0.002^a^	0.06 ± 0.002^b^	0.02 ± 0.001^c^	0.02 ± 0.001^c^	0.02 ± 0.003^c^	0.01 ± 0.000^d^
(mg/tube)	High	0.31 ± 0.018^a^	0.30 ± 0.012^a^	0.29 ± 0.011^a^	0.17 ± 0.002^b^	0.11 ± 0.004^c^	0.09 ± 0.012^c^	0.06 ± 0.006^d^

*CLA* conjugated linoleic acid, *VA* vaccenic acid, *VFA* volatile fatty acids

For each fatty acids, subscript * represents different (*P* < 0.05) compared to 0 mg/tube 22:6*n*-3 and ** represents different (*P* < 0.05) compared to 0 and 0.1 mg/tube 22:6*n*-3 at the respective time point

Within each row means with different superscripts (a-e) are significantly different (*P* < 0.05)

## Discussion

*Butyrivibrio* species are a genetically and functionally diverse group of bacteria present in gastrointestinal systems [4, 15]. Based on the mechanism of butyrate formation, this group can be classified into two subgroups: vaccenic acid-producing (low butyrate kinase activity) and stearic acid-producing (high butyrate kinase activity). Accordingly, *B. fibrisolvens* and *B. proteoclasticus* are belonging to the vaccenic acid-producing and stearic acid-producing groups respectively [16]. *Butyrivibrio fibrisolvens* D1 and *B. proteoclasticus* P18 were chosen for this study as a representative from each group. However, the type species *B. fibrisolvens* D1 showed high butyrate kinase activity which is atypical to the majority of *B. fibrisolvens* isolates [17].

Previous studies carried out with *B. fibrisolvens* and *B. proteoclasticus* in M2 medium failed to show hydrogenation of 22:6*n*-3 [2, 10]. The reason for this is probably the high concentration of 22:6*n*-3 used in these studies (50 μg/mL) which may have affected the growth of the bacteria. Wallace et al. [4] showed growth to be a prerequisite for *B. proteoclasticus* P18 in order to form stearic acid (18:0) from 18:2*n*-6. If this is also true for 22:6*n*-3 biohydrogenation, failure to grow when 22:6*n*-3 is present in previous experiments [2, 10] possibly explains the absence of 22:6*n*-3 biohydrogenation. To reduce the inhibitory effect of 22:6*n*-3 on growth, the concentration of 22:6*n*-3 in the medium can be lowered or substances which lower the toxicity of 22:6*n*-3 should be included in the medium. Harfoot et al. [18] showed that biohydrogenation was stimulated by rumen fluid particles, enabling FA biohydrogenation by ruminal bacteria. Experiments performed in our lab also showed addition of autoclaved-uncentrifuged rumen fluid stimulated the biohydrogenation of 22:6*n*-3 in mixed cultures of rumen bacteria (Escobar M, Vlaeminck B and Fievez V, unpublished results). Hence, we used autoclaved-uncentrifuged rumen fluid to prepare the growth medium. Additionally we used a lower concentration of 22:6*n*-3 than the concentration reported previously (50 μg/mL) [2, 10].

*Butyrivibrio fibrisolvens* grew at low concentrations of 22:6*n*-3 without metabolizing it. Maia et al. [2] showed that growth of *B. fibrisolvens* JW11 was not initiated until all 18:2*n*-6 was metabolized and converted to VA, such phenomenon was not observed with 22:6*n*-3. The failure of *B. fibrisolvens* D1 to metabolize 22:6*n*-3 in the current experiment might indicate they do not possess the necessary enzymes for 22:6*n*-3 biohydrogenation. However, further studies are warranted as *B. fibrisolvens* D1 is atypical to other *B. fibrisolvens* in general.

In contrast with previous reports [10], we found *B. proteoclasticus* P18 is able to hydrogenate 22:6*n*-3. To our knowledge, this is the first report demonstrating 22:6*n*-3 biohydrogenation by a pure bacterial species. Temporal changes in 22:6*n*-3, VFA and OD_600_ during the course of the incubation might indicate *B. proteoclasticus* must be growing to biohydrogenate 22:6*n*-3, with little transformation occurring during the stationary phase. Such observations were also made when demonstrating the capability of *B. proteoclasticus* P18 to form 18:0 from 18:2*n*-6 [4]. Stationary-phase bacteria were reported to be much less active in hydrogenation of 18:2*n*-6 than growing cells [4, 19]. This was explained by a limited supply of reducing equivalents once the energy sources in the medium had been depleted [4] as the conversion of CLA to VA is NADH-dependent [20]. It seems probable that a similar mechanism explains the variation in rate of disappearance of 22:6*n*-3 during the course of the incubation period. It is however not clear whether growth or activity per se is needed for biohydrogenation, or that the association of disappearance of 22:6n-3, VFA and OD_600_ is merely a reflection of changes in the culture closely associated with growth and activity (e.g. biomass production, redox potential).

The metabolism of 22:6*n*-3 resulted in the appearance of numerous compounds eluting during GC analysis between 22:0 and 22:6*n*-3. It has been speculated before that ruminal hydrogenation of 22:6*n*-3 yields intermediates with 5 or 6 double bonds, containing at least one trans double bond [1]. The position of the double bonds in the most abundant 22:5 isomer (Δ5, Δ10, Δ13, Δ16, Δ19-22:5) suggest the formation of this intermediate involves izomerisation of the cis-4 double bond and reduction of the cis-7 double bond [21]. Initial izomerisation of the cis-4 double bond of 22:6*n*-3 would result in the formation of a conjugated product (Δ5, Δ7, Δ10, Δ13, Δ16, Δ19-22:6). However, in the present series of experiments, no accumulation of products eluting in the GC chromatogram with a retention time greater than 22:6*n*-3 was observed, a region where conjugated isomers of 22:6 would be expected to elute with the polar column used in the present study. The lack of accumulation of a conjugated FA might indicate they are transient products which did not accumulate at the time of sampling. Alternatively, this might also indicate the initial product of 22:6*n*-3 metabolism is not a conjugated FA. The accumulation of 22:5 isomers produced during the initial stages of 22:6*n*-3 biohydrogenation was transient, when the initial 22:6*n*-3 concentration was low, and these isomers were subsequently hydrogenated to more saturated 22:4, 22:3 and 22:2 isomers. The existence of two isomers with identical double bond position (peak 5 and 7 as Δ13, Δ16, Δ20-22:3 and peak 9 and 11 as Δ16, Δ20-22:2) indicates double bonds in the *trans* configuration must be present. It is unclear to what extent the increases in polyenoic *trans* fatty acids may offset some of the expected benefits from the enrichment of 22:6*n*-3 in ruminant derived foods [21].

*Butyrivibrio proteoclasticus* is the only known ruminal bacterium with the capacity to biohydrogenate 18-carbon FA to 18:0. Previous incubations with rumen fluid have established that 22:6*n*-3 inhibits the complete hydrogenation of 18-carbon unsaturated FA causing trans 18:1 isomers to accumulate [8]. This is thought to be related to the toxic effects of 22:6*n*-3 on the growth and metabolic activity of *B. proteoclasticus* [22]. However, *in vivo* studies have failed to show the relationship between 18:0 flow to the duodenum and *B. proteoclasticu*s DNA [7, 23]. Based on the findings of present study we suggest that *B. proteoclasticus* probably starts to hydrogenate 22:6*n*-3 before converting 18:1 isomers to 18:0. Biohydrogenation is required to lower toxicity and therefore the reduction of products containing more double bonds are a greater priority which explains the lack of 18:1 reduction at time points where biohydrogenation of 22:6*n*-3 did occur. These findings possibly explain the lack of relationship of duodenal 18:0 to the duodenum and *B. proteoclasticu*s DNA [7, 23].

## Conclusions

*Butyrivibrio proteoclasticus* P18 is able to hydrogenate 22:6*n*-3 *in vitro*. The rate and extent of biohydrogenation depended on the initial concentration of 22:6*n*-3 and the duration of incubation. Several 22:5, 22:4, 22:3 and 22:2 isomers were identified. The products formed irrespective of the initial concentrations of 22:6*n*-3 and type of growth media suggests *B. proteoclasticus* P18 had a consistent pathway of 22:6*n*-3 hydrogenation. During the simultaneous presence of both 18:2*n*-6 and 22:6*n*-3, B*. proteoclasticus* P18 initiated 22:6*n*-3 metabolism before converting 18:1 isomers into 18:0. Under the current culture conditions, *B. fibrisolvens* D1 failed to hydrogenate 22:6*n*-3.

## Methods

### Microorganisms and cultivation

*Butyrivibrio fibrisolvens* D1 (DSM 3071) and *B. proteoclasticus* P18 were selected for this study. *Butyrivibrio fibrisolvens* D1 was purchased from DSMZ (Deutsche Sammlung von Mikroorganismen und Zellkulturen GmbH, Braunschweig, Germany) and *B. proteoclasticus* P18 was obtained from the culture collection of the Rowett Institute of Nutrition and Health (University of Aberdeen, Bucksburn, Aberdeen AB21 9SB, UK). *Butyrivibrio* medium (DSMZ: medium 704) was slightly modified: VFA mixture, haemin and glycerol were omitted from the basic medium and L-cysteine-HCl was used as the only reducing agent (0.5 g/l). The preparation method of rumen fluid and the rumen fluid/buffer ratio were also modified (Table 1).

Autoclaved-uncentrifuged or -centrifuged rumen fluids were used for the growth media preparation. Rumen fluid was collected from 3 mature wethers, fitted with a ruminal cannula, fed grass hay and a commercial grain concentrate twice a day according to their maintenance requirements. To obtain the centrifuged rumen fluid, rumen fluid collected from cannulated wethers, was filtered through a sieve with a pore size of 1 mm and then fine particles were removed from the filtrate by centrifugation at 10,000 *g* for 20 min at 4 °C. The supernatant was sterilized by autoclaving for 20 min at 121 °C and stored frozen at −20 °C. The stored rumen fluid was thawed before use and any new precipitates formed were removed by centrifugation at 12,000 *g* for 15 min at 4 °C. For autoclaved-uncentrifuged rumen fluid, rumen fluid collected from cannulated wethers was filtered through a sieve with a pore size of 1 mm and autoclaved (20 min at 121 °C), and used without further processing.

*In vitro* incubations were carried out anaerobically at 39 °C under continuous shaking in Hungate-type tubes (16 mm dia., 125 mm long; Bellco Glass, Vineland, NJ, USA) containing 9.5 ml of medium and closed with screw caps fitted with butyl rubber septa (Chemglass Life Sciences, Vineland, NJ, USA), and autoclaved for 20 min at 121 °C. Inoculum volumes were 5 % (v/v) of a fresh culture that was grown in medium 704 for 12 h (OD_600_ ≈ 1.7). Growth was determined by measuring the culture density at 600 nm (Ultraspec10, Amersham Biosciences corp., Piscataway, NJ, USA). At the end of the experiment the incubations were stopped by placing the tubes in ice water. pH was measured (Hanna instruments, Temse, Belgium) and culture contents were sampled for VFA and long chain FA (LCFA) analysis.

### Fatty acid solution

Fatty acid solutions were prepared by dispersing 200 mg of 22:6*n*-3 (Nu-check-Prep., Elysian, MN, USA) in 3.33 mL of a 0.06 M Tween-80 (Sigma Aldrich, St Louis, MO) solution. Then 0.25 mL of 3 M NaOH was added to obtain a clear solution. This solution was diluted with distilled water to achieve a final 22:6*n*-3 concentration of 10 mg/mL. The amount of Tween-80 and NaOH in all the tubes was kept the same by using a blank solution (prepared with Tween-80 and NaOH only). The required amount of the FA solution was added before autoclaving individually to each Hungate tube.

### *In vitro* experiments

Previous experiments performed with *Butyrivibrio* species in the medium containing centrifuged rumen fluid failed to hydrogenate 22:6*n*-3 [2, 10]. As such, experiments 1–5 (Table 1) were conducted using the growth medium containing autoclaved-uncentrifuged rumen fluid. In experiment 1, *B. fibrisolvens* was grown in the medium containing either 20 or 50 % (v/v) autoclaved-uncentrifuged rumen fluid. The concentration of 22:6*n*-3 used (20 μg/mL) was lower than the previously reported value (50 μg/mL) [2, 10]. All treatments were conducted with 3 replicates.

Since we saw the 22:6*n*-3 disappearance by *B. proteoclasticus* in the medium containing 50 % (v/v) autoclaved-uncentrifuged rumen fluid, additional experiments were conducted in this medium (Table 1). Influence of headspace gas (Exp. 2), incubation period (Exp. 3) and initial concentration of 22:6*n*-3 (Exp. 4) were studied in these experiments. *Butyrivibrio proteoclasticus* is the only stearic acid forming rumen bacteria so far identified. Formation of stearic acid (18:0) may be affected in the presence of 22:6*n*-3 as 22-C FA are more toxic than the 18-C FA. As such influence of 18:2*n*-6 (40 μg/mL) on biohydrogenation of 22:6*n*-3 (0, 10 and 40 μg/mL) was studied in experiment 5. All treatments were performed in duplicate (analytical replicates) with inoculum from two different culture tubes (biological replicates).

Experiments 6–8 were conducted using autoclaved-centrifuged rumen fluid (Table 1). In experiment 6, *B. fibrisolvens* was grown with different concentrations of 22:6*n*-3 and growth (culture density) was monitored during the experiment. Lag time for the growth was determined based on the time point at which the increase in OD_600_ initiated. Linoleic acid (18:2*n*-6) was used as the positive control. Treatments were performed in duplicate (analytical replicates) with inoculum from three different culture tubes (biological replicates).

In experiment 7, we saw the disappearance of 22:6*n*-3 (20 μg/mL) by *B. proteoclasticus* in the medium containing 20 % (v/v) autoclaved-centrifuged rumen fluid after 24 h incubation. Kinetics of this disappearance was studied in experiment 8 along with the growth of the bacteria by measuring increase in culture density (OD_600_) at the time of tube withdrawal. Three analytical replicates were used for each treatment.

### Analysis and calculation

For VFA analysis, 2 mL of incubation medium were collected and acidified with 200 μL of formic acid which contained the internal standard (10 mg of 2-ethyl butyric acid/mL formic acid). After 15 min centrifugation at 4 °C and 22,000 *g*, supernatant was filtered and an aliquot was transferred into a 1.5 mL glass vial. Samples were stored at 4 °C until VFA analysis using gas chromatography (HP 7890A, Agilent Technologies, Diegem, Belgium) equipped with a FID detector and a Supelco Nukol capillary column (30 m × 0.25 mm × 0.25 μm, Sigma-Aldrich, Diegem, Belgium). The temperature program was as follows: 120 °C at injection for 0.2 min; increased at 10 °C/min until 180 °C and remained at this temperature for 3 min; injector temperature: 250 °C; detector temperature 255 °C. For this temperature program, 0.3 μL was injected with a split/splitless ratio of 25:1 using H_2_ as carrier gas at 0.8 mL/min. VFA peaks were identified based on their retention times, compared to external standards (Sigma Aldrich, St Louis, MO).

The remainder of the content in the Hungate tubes after removal of 2 mL for VFA analysis (8 mL/tube) was freeze-dried for LCFA analysis and FA were methylated as described by Vlaeminck et al. [9]. Analysis of the FA methyl esters (FAME) was carried out using a gas chromatograph (HP7890A, Agilent Technologies, Diegem, Belgium) using a SP-2560 column (75 m x 0.18 mm, i.d. x 0.14 μm thickness, Supelco Analytical, Bellefonte, USA) and a flame ionization detector. The temperature program was initially 70 °C for 2 min, increasing at 15 °C/min to 150 °C, followed by a second increase at 1 °C/min up to 165 °C and holding for 12 min, followed by a third increase at 5 °C/min to 210 °C, held at 210 °C for 20 min, increased at 5 °C/min to 220 °C and held at 220 °C for 15 min. Inlet and detector temperatures were 250 and 255 °C, respectively. The split ratio was 50:1. Hydrogen was used as the carrier gas at a flow rate of 1 mL/min. Identities of peaks were determined using mixtures of methyl ester standards (GLC463, Nu-Check-Prep., Inc., Elysian, MN, USA).

Quantification of FA was based on the area of the internal standard and on the conversion of peak areas to the weight of FA by a theoretical response factor for each FA [24, 25].

### Structural analysis of fatty acid intermediates

Methyl esters not contained in commercially available standards were identified based on GC-MS analysis of DMOX derivatives prepared from FAME. Prior to preparation of DMOX derivatives, FAME were fractionated using Ag^+^-SPE columns (750 mg/6 mL, Supelco, Bellefonte, PA, USA). Columns were activated with 4 mL acetone, followed by 4 mL hexane. The FAME of selected samples, dissolved in 1 mL hexane, were loaded on the column and eluted with hexane containing increasing amounts of acetone (v/v): 6 mL (99/1), 2 × 3 mL (96/4), 2 × 3 mL (90/10), 2 × 3 mL (0/100). This was followed by elution with acetone containing increasing amounts of acetonitrile (v/v): 2 × 3 mL (98/2), 2 × 3 mL (96/4), 2 × 3 mL (94/6), 2 × 3 mL (90/10) and 2 × 3 mL (80/20). All fractions were taken to dryness in a stream of N_2_, dissolved in hexane and used for analysis of FAME by GC, as described above, and preparation of 4, 4-dimethyloxazoline (DMOX) derivatives.

DMOX derivatives of FA were prepared by using a modified procedure [26]. Briefly, FAMEs were converted into DMOX derivatives with 500 μL 2-amino-2-methyl-1-propanol under a nitrogen atmosphere at 170 °C overnight. DMOX derivatives were extracted twice with diethyl ether/n-hexane (1:1, v/v) and sodium chloride-saturated water. The organic layer was dried with anhydrous sodium sulphate for 1 h, followed by evaporation until dry under nitrogen. The DMOX derivatives were dissolved in hexane.

Identification of the DMOX derivatives of the biohydrogenation intermediates was based on electron impact ionisation spectra obtained by gas chromatography-mass spectrometry (GC-MS), using a gas chromatograph (Trace2D-GC, Thermo Electron Corporation, Waltham, MA, USA) coupled to a quadrupole mass detector (DSQII, Thermo Electron Corporation) under an ionisation voltage of 70 eV, using helium as carrier gas. The ion source and interface temperatures were maintained at 200 °C and 250 °C, respectively. The column was a SLB 5 ms capillary column (60 m x 0.25 mm, i.d. x 0.25 μm thickness, Supelco Analytical, Bellefonte, USA). The electron impact ionization spectra obtained were used to locate double bonds based on atomic mass unit (amu) distances with an interval of 12 amu between the most intense peaks of clusters of ions containing n and n-1 carbon atoms, being interpreted as cleavage of the double bond between carbon n and n + 1 in the fatty acid moiety.

### Statistical analysis

All statistical analysis was performed using SAS version 9.4 (SAS Institute Inc., Cary, NC). A least squares mean ANOVA in the GLM procedure using 22:6*n*-3 level (Exp. 1, 4, 5 and 6) or incubation time (Exp. 3 and 8) or headspace gas (Exp. 2) as factors was used to test time and treatment effects. The Tukey-Kramer test was performed to assess which treatments differed in case of multiple (> 2) treatments. The differences among means with *P* < 0.05 were considered to be statistically significant.

## Abbreviations

CLA, conjugated linoleic acid; FA, Fatty acid; FAME, Fatty acid methyl esters; OD, optical density; PUFA, polyunsaturated fatty acids; VA, vaccenic acid; VFA: volatile fatty acids.
